# Soluble Human Angiotensin- Converting Enzyme 2 as a Potential Therapeutic Tool for COVID-19 is Produced at High Levels In *Nicotiana benthamiana* Plant With Potent Anti-SARS-CoV-2 Activity

**DOI:** 10.3389/fpls.2021.742875

**Published:** 2021-12-06

**Authors:** Tarlan Mamedov, Irem Gurbuzaslan, Damla Yuksel, Merve Ilgin, Gunay Mammadova, Aykut Ozkul, Gulnara Hasanova

**Affiliations:** ^1^Department of Agricultural Biotechnology, Akdeniz University, Antalya, Turkey; ^2^Institute of Molecular Biology and Biotechnology, Azerbaijan National Academy of Sciences, Baku, Azerbaijan; ^3^Department of Virology, Faculty of Veterinary Medicine, Ankara University, Ankara, Turkey; ^4^Biotechnology Institute, Ankara University, Ankara, Turkey

**Keywords:** ACE2, SARS-CoV-2, *Nicotiana benthamiana*, plant transient expression, therapeutic agent

## Abstract

The coronavirus disease 2019 (COVID-19) pandemic, which is caused by severe acute respiratory syndrome coronavirus 2 (SARS-CoV-2) has rapidly spread to more than 222 countries and has put global public health at high risk. The world urgently needs a safe, cost-effective SARS-CoV-2 vaccine as well as therapeutic and antiviral drugs to combat COVID-19. Angiotensin-converting enzyme 2 (ACE2), as a key receptor for SARS-CoV-2 infections, has been proposed as a potential therapeutic tool in patients with COVID-19. In this study, we report a high-level production (about ∼0.75 g/kg leaf biomass) of human soluble (truncated) ACE2 in the *Nicotiana benthamiana* plant. After the Ni-NTA single-step, the purification yields of recombinant plant produced ACE2 protein in glycosylated and deglycosylated forms calculated as ∼0.4 and 0.5 g/kg leaf biomass, respectively. The plant produced recombinant human soluble ACE2s successfully bind to the SARS-CoV-2 spike protein. Importantly, both deglycosylated and glycosylated forms of ACE2 are stable at increased temperatures for extended periods of time and demonstrated strong anti-SARS-CoV-2 activities *in vitro*. The half maximal inhibitory concentration (IC50) values of glycosylated ACE2 (gACE2) and deglycosylated ACE2 (dACE2) were ∼1.0 and 8.48 μg/ml, respectively, for the pre-entry infection, when incubated with 100TCID_50_ of SARS-CoV-2. Therefore, plant produced soluble ACE2s are promising cost-effective and safe candidates as a potential therapeutic tool in the treatment of patients with COVID-19.

## Introduction

Severe acute respiratory syndrome coronavirus 2 (SARS-CoV-2) is a novel and highly pathogenic coronavirus, which caused an outbreak in the city of Wuhan, China in 2019, and then spread nationwide and spilled over to other countries, which resulted in hundreds of thousands of deaths worldwide. The General Secretary of the United Nations has described this situation as the worst crisis of humanity since World War II. Although several coronavirus disease 2019 (COVID-19) vaccines are currently available and a number of candidate vaccines are under development, the world still urgently needs effective and safe SARS-CoV-2 vaccines, antiviral, and therapeutic drugs to combat the pandemic given the emerging variant strains of the virus. The development of therapeutic drugs can be a useful and alternative approach to suppress the entry and spreading of virus. Since the angiotensin converting enzyme 2 (ACE2), the SARS-CoV-2 (COVID-19) receptor is a critical molecule in the entry process of the virus into host tissue cells, it could be a potential therapeutic agent. ACE2 is zinc containing metalloenzyme, present in most of the organs, attached to the cell membranes of cells in the lungs, heart, kidneys, arteries, and intestines (Hamming et al., 2004). ACE2 enzyme has multiple functions, the primary one being to cleave the angiotensin II to angiotensin (1–7) and to cleave the angiotensin I hormone into the vasoconstricting angiotensin II. ACE2 is a transmembrane protein that serves as a receptors for some coronaviruses, such as SARS-CoV, SARS-CoV-2, and HCoV-NL63 (Kuba et al., 2005; Li, 2013; Fehr and Perlman, 2015; Lewis, 2020; Xu et al., 2020; Zhou et al., 2020). Similar to SARS-CoV, SARS-CoV-2 has been shown to bind to its functional receptor ACE2 *via* receptor binding domain (RBD) of SARS-CoV-2 spike protein as an initial step for entry into the cell (Li et al., 2003; Tai et al., 2020). It has been demonstrated that the binding affinity between ACE2 and RBD of SARS-CoV-2 is much stronger than that of SARS-CoV (Tai et al., 2020), which explains the increased infectivity of SARS-CoV-2 versus SARS-CoV. It has been demonstrated that, ACE2 serves not only as the entry receptor for SARS-CoV or SARS-CoV-2 but can also prevent lung injuries (Imai et al., 2005; Kuba et al., 2005; Wösten-van Asperen et al., 2011; Yu et al., 2016; Zhou et al., 2020). Therefore, ACE2 has been proposed as a potential therapeutic tool to be used for SARS-CoV-2 infection (Li, 2013; Lei et al., 2020). In addition, soluble ACE2 is described as a therapeutic candidate, which could neutralize the infection by acting as a decoy (Chan et al., 2020).

Recombinant human ACE2 is proposed as a novel treatment to improve pulmonary blood flow and oxygen saturation in piglets (Colafella et al., 2019). Based on the reported pathological findings (Yang et al., 2014; Zou et al., 2014; Pang et al., 2020), it has been shown that SARS-CoV-2 is associated with lung failure and acute respiratory distress syndrome (ARDS). Pulmonary arterial hypertension (PH) is a devastating lung disease, which is characterized by high blood pressure in the pulmonary circulatory system (Schermuly et al., 2011). Taking this into consideration, the introduction of soluble recombinant human ACE2 into the human body has been proposed for the treatment of ARDS and pulmonary arterial hypertension (Zhang and Baker, 2017). It should be noted that the Phase I (NCT00886353) and Phase II (NCT 01597635) clinical trials for recombinant human ACE2 have been successfully completed. The administration of soluble recombinant ACE2 was proven to be safe and efficient for the treatment of ARDS and without significant clinical changes in healthy people, as well as in the patients with ARDS (Haschke et al., 2013; Khan et al., 2017). During the drug-taking period, there were no serious adverse events or antibodies to recombinant human ACE2 detected (Haschke et al., 2013). Recently, it was shown that recombinant human ACE2 was significantly inhibiting SARS-CoV-2 infection of Vero E6 cells (Monteil et al., 2020) and that it can neutralize SARS-CoV-2 infectivity in human kidney organoids (Wysocki, 2021), human capillary, and kidney organoids (Monteil et al., 2020). It was previously proposed that SARS-CoV may deregulate a lung protective pathway (Imai et al., 2005; Kuba et al., 2005). Thus, recombinant ACE2 may not only reduce lung damage, but could also block at an early stage the entry of SARS-CoV-2 infections in target cells. Therefore, the development of a cost-effective, safe, and functionally active recombinant ACE2 could be very important in the treatment of patients with COVID-19. Recent studies have shown that the plant expression systems are promising expression platforms for the rapid, safe, and cost-effective production of various recombinant proteins. Plant expression systems have a number of advantages compared with other expression systems currently used, and have the ability to accumulate hundreds of milligrams of target protein per kilogram of biomass in less than a week. This system has been successfully used for rapid and cost-effective production of a variety of recombinant proteins, such as vaccine candidates Mamedov et al., 2012, 2016, 2017, 2019a,2019b, 2021) and complex mammalian proteins, and enzymes, such as Furin and Factor IX (Mamedov et al., 2019b). Notably, transplastomic technology human ACE2 was produced in plant chloroplasts (Shil et al., 2014). It was demonstrated that the delivery of human ACE2 (fused with non-toxic cholera toxin subunit B, CTB) by oral gavage in mice resulted in increased circulating and retinal levels of ACE2 and reduced eye inflammation (Shil et al., 2014). Expression of ACE2 fused with the Fc region of human IgG1 in *Nicotiana benthamiana* plant using a transient expression platform has been recently reported (Siriwattananon et al., 2021). The expression level of ACE2 fusion protein was 100 mg/kg of plant leaves. It was reported that this plant-produced fusion protein exhibited a potent anti-SARS-CoV-2 activity *in vitro*.

In addition, the ACE2 enzyme was produced in various mammalian cells, such as HEK293, CHO, insect cells, and commercially available^1, 2, 3^. Although about 60–70% of all recombinant pharmaceuticals are produced in mammalian cells (McDonald, 2011; Gupta et al., 2016), however, large-scale culture of mammalian cells is more expensive and technically challenging compared with yeast, bacterial, and other cells (Araki et al., 2012; Berlec and Strukelj, 2013; Korn et al., 2020). In addition, there is a risk of contamination of mammalian pathogens in recombinant proteins produced using the mammalian expression systems. Here we describe for the first time the engineering, expression, and production at a high level of truncated, soluble form of ACE2 in *N. benthamiana* plant using a transient expression system. The expression levels of both glycosylated and *in vivo* Endo H deglycosylated forms are ∼0.75 g/kg leaf biomass. After the Ni-NTA single step, the purification yields of recombinant plant produced ACE2 protein glycosylated and deglycosylated forms calculated as ∼0.4 and ∼0.5 g/kg of leaf biomass, respectively. Expression of ACE2 proteins and purification procedures can be optimized to increase their purification yield. Our results demonstrated that the plant-produced soluble ACE2 proteins bind successfully to the RBD of the SARS-CoV-2 spike protein and inhibit SARS-CoV-2 infection *in vitro*. In addition, our results demonstrate that both glycosylated ACE2 (gACE2) and deglycosylated ACE2 (dACE2) proteins are stable at increased temperatures for prolonged periods of time.

## Materials and Methods

### Cloning, Expression, and Screening of Recombinant ACE2 in *N. benthamiana* Plants

The sequences of ACE2 (without a transmembrane domain and cytoplasmic tail) were optimized for expression in *N. benthamiana* plants and synthesized by Biomatik (Biomatik Corporation, ON, Canada). To express ACE2 in *N. benthamiana* plants, the signal peptide (amino acids 1–17) was replaced with the *Nicotiana tabacum* PR-1a signal peptide (MGFVLFSQLPSFLLVSTLLLFLVISHSCRA). In addition, the endoplasmic reticulum (ER) retention signal (KDEL) and the His6 tag coding sequences were added to the C-terminus. The constructed ACE2 gene was inserted into the pEAQ binary expression vector (Sainsbury et al., 2009) to obtain pEAQ-ACE2- His6-KDEL. pEAQ-ACE2-His6-KDEL plasmid was introduced into the *Agrobacterium tumefaciens* strain (AGL1). AGL1 carrying the pEAQ-ACE2-His6-KDEL plasmid was then infiltrated into 6–7-week-old *N. benthamiana* plants. To produce dACE2, ACE2 gene was *in vivo* co-expressed with Endo H (Mamedov et al., 2017). To confirm the expression of His6 tagged ACE2 protein variants, a leaf tissue was harvested at different dpi (day post infiltration) and homogenized in three volumes of extraction buffer (20 mM sodium phosphate, 150 mM sodium chloride, pH 7.4) as described previously (Mamedov et al., 2019a). To quantify the expression levels of ACE2 variants, the plant cell extract was filtered through Miracloth and then centrifuged at 20,000 × *g* for 25 min at 4°C and then, clear extract was analyzed by western blot or ELISA using purified anti-His tag mouse mAb (Cat. no. 652505, BioLegend, CA, United States) or purified anti-human ACE2 antibody (Cat. no. 375801, BioLegend, CA, United States). For ELISA, plates were coated with 50 μl of diluted supernatants containing (i) gACE2 or (ii) dACE2 or (iii) commercial recombinant human ACE2, expressed in CHO cells (human ACE2, amino acids Gln18—Ser740, accession: NM_021804.1) as a standard protein and incubated overnight at 4°C. After blocking for 2 h at 37°C, purified anti-human ACE2 antibody (Cat. no. 375801, BioLegend, CA, United States) was added into each well and incubated at 37°C for 1 h, and then, the plate was washed three times with PBST solution [1x phosphate-buffered saline (PBS) containing 0.05% Tween-20, 200 μl/well]. After the washing, an HRP Goat anti-rat IgG antibody (BioLegend, cat. no. 405405) was added and incubated at 37°C for 1 h. After washing the plate three times with 1X PBST washing solution, 200 μl of Substrate Solution (o-Phenylenediamine dihydrochloride, Sigma, Ronkonkoma, NY, United States) was added to each well and then, incubated for 30 min in the dark, at room temperature. After the incubation period, the plate was read at 450 nm on a multi-well plate reader.

*Nicotiana benthamiana* seeds (research accession: RA-4) were gifted from Dr. Amit Mitra, University of Nebraska–Lincoln, United States. Experimental research and studies on plants, such as the collection of plant material, comply with the relevant institutional, national, and international guidelines and regulations.

### Purification of Recombinant ACE2 From *N. benthamiana* Plants

To produce the ACE2 protein (both gACE2 and dACE2) in *N. benthamiana*, plants were infiltrated with ACE2 (glycosylated) or ACE2 + Endo H (deglycosylated) genes and harvested at 6 dpi. For purification, 20 g of frozen plant leaves from each variant, infiltrated with the ACE2 gene, were grounded in an extraction buffer with three times volume of plant weight and the extract was centrifugated for 20 min at 4°C at 13,000 × *g*. The supernatant was loaded onto a disposable polypropylene column (Pierce) with 1 ml HisPur*™* Ni-NTA resin equilibrated with 10 column volume binding buffer (20 mM sodium phosphate, 300 mM sodium chloride, 10 mM imidazole, pH 7.4), by gravity-flow chromatography. The column was washed with 10–15 column volumes (CV) of wash buffer (20 mM sodium phosphate, 300 mM sodium chloride, 25 mM imidazole; pH 7.4) until reaching the baseline. Proteins were eluted with 10 CV of elution buffer (20 mM sodium phosphate, 300 mM sodium chloride, 250 mM imidazole; pH 7.4). Elution fractions were collected as 0.5 ml/Eppendorf and protein concentrations in the eluted fractions were measured by BioDrop. According to the concentration, the combined fractions were concentrated, and buffer exchanged against PBS with a 10K MWCO Millipore concentrator (Cat No: UFC801096, Merck, NJ, United States) to a final volume of 1.2 ml. The concentrated protein was stored at −80°C until used.

### Gel Filtration

Gel filtration of plant produced gACE2 or dACE2 proteins was performed with ÄKTA start on a C 10/40 column (cat. no. 19-5003-01, GE Healthcare, Chicago, IL, United States), packed with Sephacryl^®^ S-200 HR (cat. no. 17-0584-10, GE Healthcare, Chicago, IL, United States). The column was equilibrated with 50 mM phosphate buffer, containing 150 mM NaCl, pH 7.4 and then, 0.5 mg His-tag affinity column purified gACE2 or dACE2 proteins, were loaded onto a column. Bovine serum albumin (BSA, cat. no. A2153-10G, Sigma, ∼66 kDa, monomer, MO, United States) or plant produced glycosylated PA83 (∼90 kDa, monomer, Chichester et al., 2013) proteins were also loaded into column as control proteins. Eluted fractions were combined and concentrated, and buffer exchanged against PBS and concentrated with a Millipore 10K MWCO Amicon Ultra 4 concentrator (Cat. no. UFC8010, Merck, NJ, United States) and analyzed on sodium dodecyl-sulfate polyacrylamide gel electrophoresis (SDS-PAGE) in reducing and non-reducing conditions.

### Study of Binding Activity of Plant Produced Recombinant ACE2 Protein With Commercial or Plant Produced Spike Protein

Binding activity of plant produced recombinant ACE2 proteins with commercial or plant produced RBD of spike proteins of SARS-CoV-2 was performed by using ELISA. A 96-well plate (Greiner Bio-One GmbH, Germany) was coated with 100 ng of plant produced RBD (R319-S591) (Mamedov et al., 2020, 2021) or commercial insect RBD of SARS-CoV-2 (RBD, His Tag, Arg319-Phe541, MM∼ 25 kDa, Cat. no. MBS2563882, MyBioSource, CA, United States) in 100 mM carbonate buffer and left overnight. The next day, wells were blocked with a blocking buffer (0.5% I-block in PBS) for 2 h at room temperature. After blocking, various concentrations of plant produced gACE2 and dACE2 proteins (100–2000 ng) were added into wells and incubated for 2 h at 37°C. After 2 h, purified anti-His tag mouse mAb (Cat. no. 652505, BioLegend, CA, United States) or purified anti-human ACE2 antibody (Cat. no. 375801, BioLegend, CA, United States) was added into each well. The plate was washed three times with a blocking solution (200 μl/well). After washing, wells were incubated with anti-mouse HRP−IgG antibody (Cat. no. 405306, BioLegend, CA, United States) or anti-human IgG + HRP antibody (Cat. no. MBS440121, MyBioSource, San Diego, CA, United States). The plate was washed three times with washing solution (200 μl/well for 5 min). Then, 200 μl of Substrate Solution (Sigma) was added to each well. Afterward, the plate was incubated in the dark for 30 min at room temperature. After the incubation period, the plate was read at 450 nm on a multi-well plate reader.

### Stability Assessments of Different Variants of ACE2

Stability assessments of different variants of ACE2 were performed using a similar procedure as described previously (Mamedov et al., 2017, 2019b). Plant produced glycosylated and deglycosylated variants of ACE2 were diluted to 1.0 mg/ml with PBS and were aliquoted into low-binding tubes. Proteins were then incubated at 37°C for 24, 48, 72, 96, 120, and 144 h. After incubation, the samples were analyzed by SDS-PAGE and ELISA. For SDS-PAGE analyses, the samples were mixed with SDS loading dye (5X) and stored at −20°C until used. All the samples were then run on SDS-PAGE. The degradation of ACE2 variants were quantified using highly sensitive Gene Tools software (Syngene Bioimaging, United Kingdom) and ImageJ software^4^. Plant produced gACE2 or dACE2 proteins, which were incubated at 37°C for 72 or 144 h were used for ELISA to analyze their binding affinity to commercial S protein (Com S) or plant produced dRBD (Mamedov et al., 2021).

### Sodium Dodecyl-Sulfate Polyacrylamide Gel Electrophoresis and Western Blot Analysis

Sodium dodecyl-sulfate polyacrylamide gel electrophoresis and western blot analyses, of plant produced His6-tagged, gACE2 dACE2 were performed as described previously (Mamedov et al., 2019b,^2021^). For SDS-PAGE analyses, purified, plant produced ACE2 proteins were loaded into wells, and run on 8 or 10% SDS-PAGE. For western blot analysis, proteins were first run on 10% SDS-PAGE and then transferred onto a PVDF (polyvinylidene fluoride) membrane (Millipore, Billerica, MA, United States) and blocked with a 0.5% Blotting-Grade Blocker [(Cat No: #1706404, Bio-Rad, California, United States) and then detected using anti-His mAb (Cat. No. 652505, BioLegend, CA, United States)]. ACE2 proteins (commercial and plant produced ACE2) have been detected using ACE2 specific, a purified anti-human ACE2 antibody (Cat. No. 375801, BioLegend, CA, United States). In the western blotting, the images were taken using a highly sensitive GeneGnome XRQ Chemiluminescence imaging system.

### Anti-SARS-CoV-2 Activity of Plant Produced ACE2s

Anti-severe acute respiratory syndrome coronavirus 2 potential of ACE derivates was monitored *in vitro*. To do this, we analyzed the blocking capacity of plant produced gACE2 or dACE2 variants at different concentrations. Purified dACE2 and gACE2 (initial concentrations were 3.055 and 2.542 mg/ml, respectively) were five-fold diluted in high glucose Dulbecco’s modified eagle medium (DMEM) in a U-bottomed plate. After being combined with an equal volume (100 μl) of 100TCID50 virus, the mixtures were incubated at room temperature for 30 min. A total of 150 μl incubated mixture was then inoculated on Vero E6 Cells grown in a 96-well flat-bottomed tissue culture plate (Greiner, Germany). The highest concentration (6 μg/ml) of dACE2 and gACE2 without the virus was involved as a toxicity control, and serum-free High glucose DMEM was added to each plate as a cell control. A total of 75 μl 100TCID_50_ SARS-CoV-2 Ank1 virus was used as virus control. All tests were performed in a quadruplicate. The plates were incubated at 37°C in a humidified incubator with a 5% CO_2_ atmosphere until virus control wells had adequate cytopathic effect (CPE) readings. The test was evaluated when the virus control wells showed 100% CPE at daily microscopy. To do precise calculations based on the optical density (OD) values, cells were fixed with 10% formaldehyde for 30 min and subsequently stained with crystal violet (CV–0.075% in ethanol) for 20 min. The dye washed away by repeated washing and retained CV was released by adding 100 μl ethanol (70%). Ten minutes after, the plate was read on ELISA reader using 295 nm filter (Multiskan Plus, MKII, Finland). TCID50 titer was determined as described before (Reed and Muench, 1938).

## Results

### Engineering, Production, and Purification of Recombinant ACE2s in *N. benthamiana* Plants

**FIGURE 1:**
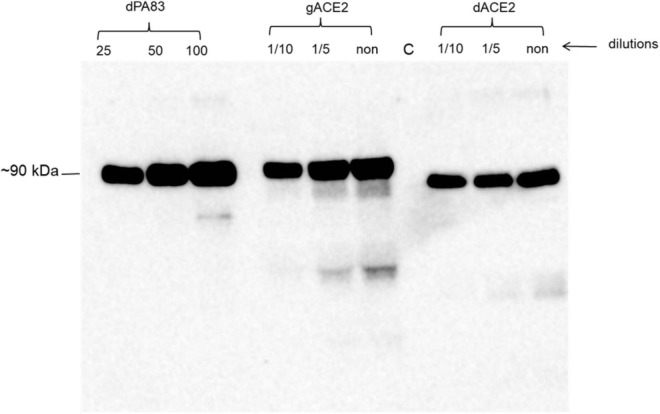
A Western blot analysis of human ACE2s, produced *in Nicotiana benthamiana* plants. dACE2: angiotensin-converting enzyme 2 (ACE2) co-expressed with bacterial Endo H, produced in *N*. *benthamiana*, different concentration (dilutions) of crude extract were loaded into wells*;* gACE2: western blot analysis of human ACE2, produced in *N*. *benthamiana* plants; different concentration (dilutions) of crude extract were loaded into wells; C-undiluted crude extract from non-infiltrated *N*. *benthamiana*, was loaded into well; gPA83: 25, 50, and 100 ng of purified plant produced dPA83 of *Bacillus anthracis*, loaded as a control protein to quantify the expression levels of ACE2 and ACE2 proteins. Purified anti-His Tag antibody (Cat. No. 652502, BioLegend) was used as primary and mouse IgG used as secondary antibodies to detect ACE2 proteins.

**FIGURE 2:**
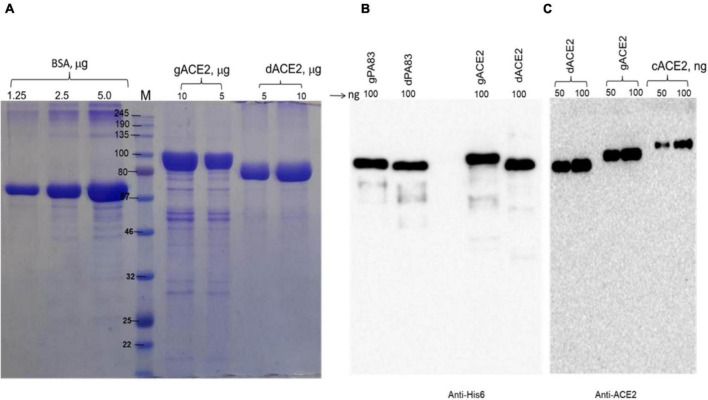
Sodium dodecyl-sulfate polyacrylamide gel electrophoresis (SDS-PAGE) **(A)** and Western blot **(B,C)** analysis of plant produced, Ni-NTA resin purified gACE2 or dACE2 proteins. Glycosylated and deglycosylated plant produced ACE2 proteins were purified from *N. benthamiana* plant using HisPur*™* Ni-NTA resin. gACE2: 5 or 10 μg purified gACE2 protein was loaded into well; dACE2: 5 or 10 μg purified dACE2 proteins were loaded into well. Bovine serum albumin (BSA) standards: 1.0, 2.5, and 5.0 μg BSA protein was loaded as a standard protein. **(B)** Membrane was probed with anti-His6 antibody. gPA83 (plant produced glycosylated protective antigen of *B. anthracis*, MM ∼100 kDa) and dPA83 (deglycosylated protective antigen of *B. anthracis*, MM ∼90 kDa) proteins were used as a standard. **(C)** Membrane was probed with a purified anti-human monoclonal ACE2 antibody (Cat. No. 375801, BioLegend, CA, United States). cACE2: recombinant human ACE2, amino acid (Gln18—Ser740) (Accession: NM_021804.1) was expressed in CHO cells as His tagged protein (cat. no. 792004, BioLegend, CA, United States). The image was taken using a highly sensitive GeneGnome XRQ Chemiluminescence imaging system.

We engineered and produced a truncated version of human ACE2 in *N. benthamiana* plant. To understand the role of glycosylation, we produced both glycosylated and de-glycosylated variants of ACE2 protein in *N. benthamiana* plant. Figure 1 demonstrates the confirmation of the production of glycosylated and deglycosylated variants of ACE2 in *N. benthamiana* by western blot analysis. The *N. benthamiana* leaf samples were harvested at different post-infiltration days (dpi) and expression levels of glycosylated and deglycosylated variants of ACE2 reached the maximum level at 6 dpi. For purification, a vacuum infiltration was used for large-scale production of glycosylated and de-glycosylated variants of ACE2. Glycosylated and deglycosylated variants of ACE2 were purified using HisPur*™* Ni-NTA resin. The purification yields of recombinant plant produced glycosylated or deglycosylated forms after Ni-NTA column were ∼0.4 and ∼0.5 g/kg of leaves, respectively. The purity of glycosylated and deglycosylated variants of ACE2 enzyme was higher than 90 or 95%, for glycosylated or deglycosylated, respectively, as estimated on SDS-PAGE using BSA as a standard protein (Figure 2A) and western blot analysis using plant produced, purified deglycosylated His tagged-PA83 as a standard protein (Figure 2B). Based on SDS-PAGE, under reducing conditions, molecular masses were ∼80 and ∼90 kDa for dACE2 and gACE2, respectively (Figure 2).

**FIGURE 3:**
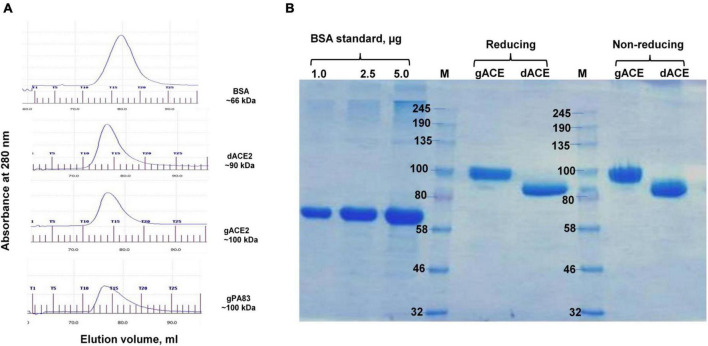
Gel filtration chromatography **(A)** and SDS-PAGE **(B)** of plant-produced gACE2 or dACE2 proteins, eluted from Sephacryl^®^ S-200 HR column. **(A)** Profiles of BSA, plant-produced gACE2, dACE2, and PA83 proteins, eluted from Sephacryl^®^ S-200 HR column. The column was equilibrated with 50 mM phosphate buffer (with 150 mM NaCl, pH 7.4). BSA, plant-produced dACE2, gACE2, and gPA83 proteins, purified using His-tag affinity chromatography, were loaded onto columns. Gel filtration was performed with ÄKTA start using C 10/40 column (cat. no. 19-5003-01, GE Healthcare, Chicago, IL, United States), packed with Sephacryl^®^ S-200 HR (cat. no. 17-0584-10, GE Healthcare). gPA83: plant produced, glycosylated PA83 of *B. anthracis*, produced in our laboratory (Mamedov et al., 2017, 2019a). **(B)** SDS-PAGE analysis of plant-produced gACE2 and dACE2 proteins eluted from Sephacryl^®^ S-200 HR column, in reducing and non-reducing conditions as indicated. Lanes were loaded with 2.5 μg gACE2 or dACE2.

Plant-produced gACE2 and dACE2 proteins purified using HisPur*™* Ni-NTA resin were subjected to size exclusion chromatography. Both gACE2 and dACE2 were eluted as single picks from Sephacryl S-200 column (Figure 3A), with elution volumes of 15.62 and 15.86 ml, respectively, and were present as monomers (Figure 3A) as eluted between gPA83 (monomer, ∼90 kDa) and BSA (monomer, ∼66 kDa). No dimerization or aggregation was observed for plant produced gACE2 and dACE2 proteins (Figure 3B). It is worth noting that although ACE2-Fc has been proposed as a dimer (Siriwattananon et al., 2021), it is very difficult to draw such a conclusion without size exclusion chromatography. Since the high molecular mass of ∼ 250 kDa, which was observed in SDS-PAGE is larger than the size of the dimer (∼ 200 kDa), the presence of a high molecular mass protein band is probably due to aggregation. Plant produced gACE2 and dACE2 migrate as single proteins under both reducing and non-reducing conditions (Figure 3B). Notably, human ACE2, amino acid (Gln18–Ser740, accession no.: NM_021804.1), which was expressed in CHO cells was shown to migrate at approximately 100 kD in SDS-PAGE, under reducing and non-reducing condition, as described in product details (cat. no. 792004, BioLegend, CA, United States). After Sephacryl S-200 column chromatography, the purity of glycosylated and deglycosylated variants of ACE2 enzyme was higher than 95%, as determined by Coomassie stained SDS-PAGE.

### Binding Affinity of Plant Produced Recombinant ACE2 Protein With Spike Protein

**FIGURE 4:**
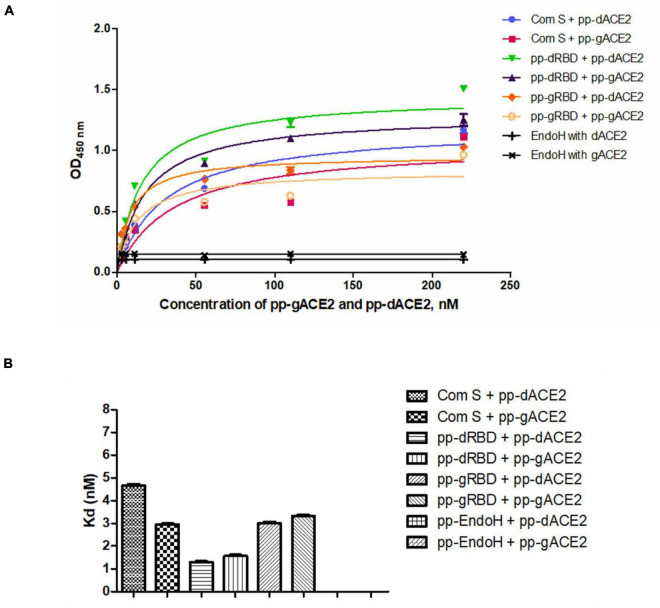
Binding activity of plant produced glycosylated or deglycosylated variants of ACE2 with commercial or plant produced, glycosylated or deglycosylated forms of spike proteins (Flag tagged). Commercial or plant-produced spike protein was coated with an ELISA plate at a concentration of 200 ng/well. Different concentration of plant produced, Sephacryl^®^ S-200 HR column purified, gACE2 or dACE2 proteins (His-tagged) were added. Purified anti-human monoclonal ACE2 antibody (Cat. No. 375801, BioLegend) was used as a primary and rat IgG used as secondary antibodies. Com S: commercial Spike protein, active Recombinant 2019-nCoV Spike Protein, RBD, His Tag, produced in Baculovirus-Insect Cells, Cat. no. MBS2563882); pp-gRBD: plant produced glycosylated Receptor Binding Domain of Spike protein (Mamedov et al., 2021); pp-dRBD: plant produced deglycosylated RBD (Mamedov et al., 2021); pp-gACE2: plant produced gACE2; pp-dACE2: plant produced Endo H *in vivo* dACE2; Endo H, plant produced Flag-tagged protein was used as negative control. **(A,B)** Graph for binding affinity between pp-gACE2 and pp-dACE2 to spike protein variants. **(A)** A graph was plotted with non-linear regression analysis in GraphPad software. Points refer to absorbance for each sample dilutions and lines were plotted according to Kd value. **(B)** Column bar graph of Kd values determined with non-linear regression analysis in GraphPad software.

Plant produced gACE2 and dACE2 proteins, purified on a Sephacryl^®^ S-200 HR column, were used to assess the binding activity. The binding activity of plant produced recombinant ACE2 protein was confirmed by measuring the binding activity of ACE2 with commercially available spike protein or plant produced RBD of spike protein of SARS-CoV-2. The results presented in Figure 4 demonstrate that plant produced gACE2s and dACE2s successfully bind to commercial spike protein or plant produced RBD of spike protein of SARS-CoV-2. Equilibrium dissociation constant (Kd) values (Figure 4B) ranged from 1.287 ± 0.0317 nM (plant produced dRBD and plant produced dACE2) to 4.678 ± 0.0367 nM (com S and plant produced dACE2), and a comparable stronger binding effect was observed between plant produced dRBD and dACE2 proteins (1.287 ± 0.0317 nM). This Kd value, determined by ELISA in this study is comparable to Kd reported for hACE2—spike protein of SARS-CoV-2 (1.2 ± 0.1), determined using Blitz (Walls et al., 2020). Notably, SARS-CoV-2-RBD binding to hACE2, determined by ELISA was reported to be 5.09 nM (Yi et al., 2020), which is comparable with Kd determined using Blitz, 2.9 nM, reported by Shang et al. (2020).

### Stability Assessment of Plant Produced ACE2s

**FIGURE 5:**
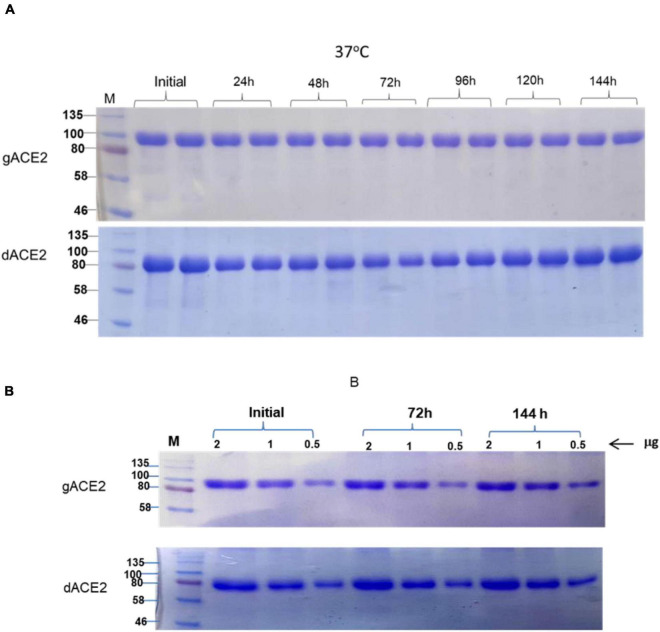
Stability assessment of plant produced gACE2 and dACE2 proteins. **(A)** Plant produced, Ni-NTA resin column purified gACE2 or dACE2 variants were incubated at 37°C for 24, 48, 72, 96, 120, and 144 h, and analyzed in SDS-PAGE. Lanes were loaded with 5.0 μg gACE2 or dACE2. **(B)** Plant produced, Ni-NTA resin column purified gACE2 or dACE variants were incubated for 72 and 144 h, and different amounts (0.5, 1.0, and 2.0 μg) from each sample were analyzed in SDS-PAGE M: a color prestained protein standard (New England Biolabs, MA, United States).

**FIGURE 6:**
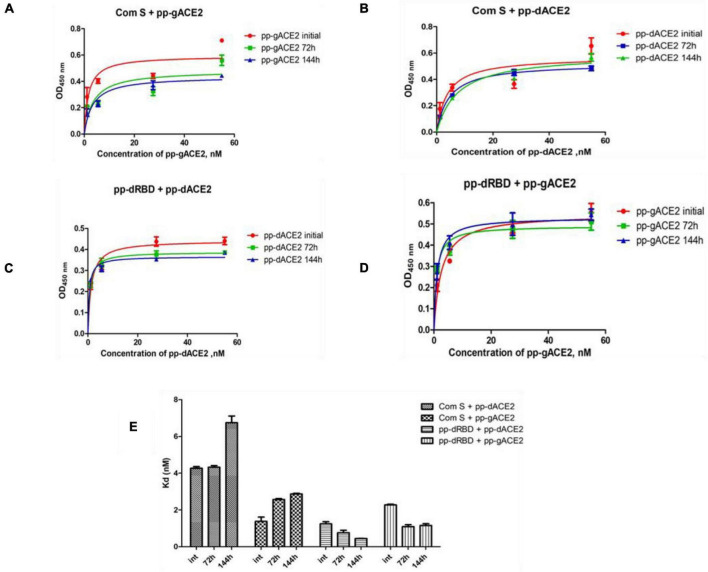
Binding affinity of plant produced gACE2 and dACE2 proteins after incubation at 37°C for 72 and 144 h. Plant produced gACE2 or dACE2 proteins incubated at 37°C for 72 and 144 h were used for ELISA to analyze binding affinity to commercial S-protein (Com S) or dRBD. **(A–D)** A Graph was plotted with the non-linear regression analyses in GraphPad software. Points refer to absorbance for each sample dilution and lines were plotted according to Kd value. **(E)** Column bar graph of Kd values determined with the non-linear regression analyses in GraphPad software.

The stability of plant produced, Ni-NTA resin column purified, glycosylated and *in vivo* deglycosylated forms of ACE2 were examined after incubation at 37°C for a prolonged time-period: 24, 48, 72, 96, 120, and 144 h (Figure 5A). An analysis by SDS-PAGE showed that plant produced gACE2 had almost no degradation at 37°C for 144 h and degradation of *in vivo* Endo H dACE2 at the same condition was less than 5%. To assess the absence of degradation, different amounts of each ACE2 sample were loaded for 72 or 144 h time points. These results were shown in Figure 5B. Stability assessment was further evaluated by using an ELISA binding study. We conducted the binding affinity study of plant produced glycosylated and deglycosylated ACE2 proteins, incubated at 37°C for 24, 48, 72, 96, 120, and 144 h, with commercial S protein and plant produced dRBD (Figures 6A–D). The Kd values were calculated using the GraphPad Prism 5.0 software (Figure 6E). Although the binding affinity of gACE2 and dACE2 proteins that were incubated at 37°C for 72 or 144 h was reduced for the commercial Spike protein, it did not change significantly for plant-produced dRBD. The difference in Kd values could be explained by several factors, such as a different glycosylation status, different tags (FLAG-tagged of plant produced RBD vs. His tagged of commercial insect RBD) and different amino sequences (R319-S591 of plant produced RBD vs. Arg319-Phe541 of commercial insect RBD), and plant produced and commercial insect RBD. It should be noted that, although the baculovirus-insect cell system is limited by its inability to produce complex N-glycans, recombinant proteins produced in some insect cell lines may contain core α1,3-linked fucose residues (Shi and Jarvis, 2007). Thus, based on SDS-PAGE and ELISA data, it can be concluded that plant-produced gACE2s and dACE2s are stable at increased temperatures for prolonged periods of time.

### Anti-SARS-CoV-2 Activity of Plant Produced ACE2s

**FIGURE 7:**
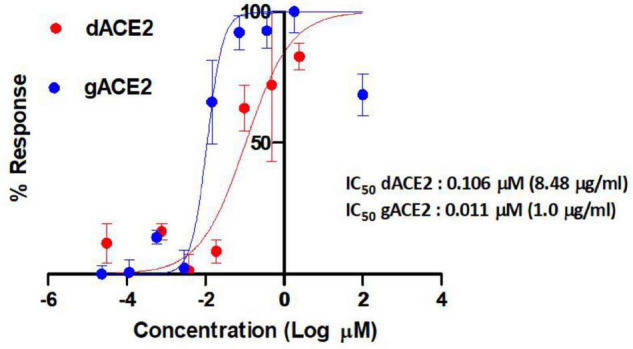
Apparent activities of two distinct ACE2 derivatives produced in plants to RBDs plotted against half maximal inhibitory concentration (IC50) of authentic SARS-CoV-2 neutralization. The IC50 values of the gACE2 (glycosylated) and dACE2 (deglycosylated) were calculated using normalized optical density (OD) data obtained from quadruplicated test dilutions in GraphPad Prism v8.2 software (GraphPad). The OD values from untreated (cell control) wells were used as normalization standards. A non-linear regression analysis was performed using log (inhibitor) versus normalized response-variable slope. The *R* square values were recorded as 0.86 and 0.89 for dACE2 and ACE2, respectively. ACE2, gACE2, glycosylated ACE2; dACE2, plant produced deglycosylated ACE2.

Anti-severe acute respiratory syndrome coronavirus 2 activities of plant produced glycosylated and deglycosylated forms were evaluated. Figure 7 demonstrates apparent neutralization activities of plant produced recombinant truncated gACE2 and dACE2 variants against authentic SARS-CoV-2 in the pre-infection phase. The half maximal inhibitory concentration (IC50) values for gACE2 and dACE2 were ∼1.00 μg/ml (0.011 μM) and 8.48 μg/ml (0.106 μM), respectively, when they were mixed with 100TCID50 of SARS-CoV-2 (Figure 7). It should be noted that in the test, the highest concentration (6 μg/ml) of gACE2 or dACE2, was non-toxic to cells.

## Discussion

The new COVID-19 disease, which is currently responsible for the pandemic, created a huge global human health crisis with significant negative impacts on health and economies worldwide. Given the high rates of morbidity and mortality associated with COVID-19, there is an urgent demand for the development of effective, safe, and affordable therapeutics, vaccines, and inhibitors to control the epidemic. A number of studies performed with SARS-CoV and SARS-CoV-2 have highlighted the ACE2 enzyme as a potential therapeutic tool and alternative treatment option in patients with COVID-19. Therefore, the aim of this study is to lead to the production of an affordable, safe, and functional active recombinant human ACE2 using the plant transient expression system for use in the treatment of COVID-19. A plant expression system is a promising expression platform for a cost-effective, fast, and safe production of a variety of recombinant proteins, vaccines, antibodies, therapeutic proteins, and enzymes (Conrad et al., 1998; Gleba et al., 2005; Tusé, 2011; Tusé et al., 2014, 2020; Walwyn et al., 2015; Nandi et al., 2016; Alam et al., 2018; McNulty et al., 2020; Shanmugaraj et al., 2020; Maharjan and Choe, 2021). Plant expression systems have several advantages over other expression systems currently in use, such as rapid and high production with the ability to accumulate grams of target protein per kilogram of biomass in less than a week (Gleba et al., 2005; Sainsbury et al., 2009; Holtz et al., 2015; Maharjan and Choe, 2021). This system has been successfully used for the production of functional active complex proteins, such as full length Pfs48/45 of *Plasmodium falciparum* (Mamedov et al., 2019b), human Furin, Factor IX (Mamedov et al., 2019a), glycohormone erythropoietin (Grosse-Holz et al., 2018), latent transforming growth factor-beta (TGF-beta) (Wilbers et al., 2016), human epidermal growth factor (Hanittinan et al., 2020), PA83 of *B. anthracis* (Mamedov et al., 2016, 2017), HIV gp140, other viral glycoproteins (Margolin et al., 2018, 2020a), and antigens against influenza, Ebola, dengue, rotavirus, and norovirus (Mett et al., 2008; Landry et al., 2014; Rybicki, 2014; Pêra et al., 2015; Takeyama et al., 2015). The goal of this study is to lead to the development of a safe, cost effective plant produced ACE2 enzyme with anti-SARS-CoV-2 activities to use in the treatment of COVID-19. ACE2 is zinc-metalloproteinase type 1 transmembrane protein with molecular mass of 120 kDa and composed of 805 amino acids. A 3D structure analysis of ACE2 protein has revealed that the enzyme molecule contains a signal peptide sequence (1–17 aa) and an extracellular sequence (18–740) which contains an active carboxy peptidase domain, a transmembrane domain (aa 741–761), and a cytoplasmic domain (aa 762–805). The ACE2 enzyme has been intensively studied since 2002 as it was identified as a cellular receptor for the SARS-CoV, HCoV-NL63, and SARS-CoV-2 coronaviruses.

In this study, for the first time, we engineered and expressed a truncated form of human ACE2 gene in *N. benthamiana* plant. Spike-glycoprotein of SARS-CoV-2 has 22 potential N-glycosylation sites. Human ACE2 is a glycoprotein, which has seven potential N-glycosylated sites. The virus (SARS-CoV-2) and receptor (ACE2) binding affinity on the surface of human cells could be a critical step in the viral entry into the susceptible cells. To understand the role of N-glycosylation, we produced both glycosylated and deglycosylated variants of ACE2 protein in *N. benthamiana* plant. DACE2 variant was produced using the *in vivo* deglycosylation strategy, by co-expression of human ACE2 with bacterial Endo H, which we have recently developed (Mamedov et al., 2017). The expression level of glycosylated and deglycosylated variants was about ∼0.75 g/kg of plant biomass. In this study, ACE2 protein was expressed using pEAQ vector (Sainsbury et al., 2009). In this vector, transgene expression was controlled by the constitutive Cauliflower mosaic virus (CaMV) 35S promoter and translation enhancer sequences derived from Cowpea mosaic virus (CPMV) RNA-2. Using this vector, the yields of up to 1.5 g/kg were achieved in *N. benthamiana* plant (Sainsbury et al., 2009). In this study, the ACE2 proteins (glycosylated and deglycosylated vesicles) were produced as a truncated version (no transmembrane and cytoplasmic tail), as a non-fusion protein, and as a native-like protein. For expression in plants, our approach was engineering of the ACE2 sequence by fusing C-terminus of the ER retention signal (KDEL) to produce of ACE2 in ER, which is very well equipped with the molecular chaperones and folding assistants for protein folding and post-translational modifications of target proteins (Stronge et al., 2001; Margolin et al., 2020b). The other advantages of expression in ER are that ER provide high level expression of target glycoproteins with high mannose N-glycan structure that is common in humans, yeast, and plants. The targeting of the protein to the ER provided a high level of production of a number of complex and difficult to-express proteins in *N. benthamiana* plant, such as full length Pfs48/45 or Pfs230 of *P. falciparum* (Farrance et al., 2011; Mamedov et al., 2019b), heptameric form of protective antigen of *B. anthracis*, human Furin and Factor IX (Mamedov et al., 2019a), and RBD of spike protein of SARS-CoV-2 (Mamedov et al., 2021); human Interleukin 6 (Nausch et al., 2012); hemagglutinin from a range influenza viruses (Chichester et al., 2009); monoclonal antibodies (Vézina et al., 2009; Holtz et al., 2015); HIV gp140 (Margolin et al., 2020a). In addition, other factors that can provide more target accumulation are Agro optimization (Shamloul et al., 2014; Hanittinan et al., 2020; Kaur et al., 2021), especially using appropriate target specific Agrobacterium strains (Norkunas et al., 2018), activation of agrobacteria with MES (2-morpholinoethanesulfonic acid), MgCl_2_, Acetosyringone (Shamloul et al., 2014; Kaur et al., 2021), and to control humidity, temperature, and light during plant growth (Shamloul et al., 2014). In general, these factors allowed the production of the recombinant soluble form of ACE2 at a high level in the *N. benthamiana* plant. It should be noted that the production of soluble human ACE2 as a fusion protein with immunoglobulin Fc domain has been recently reported (Siriwattananon et al., 2021). The production of target proteins with Fc domain can be a useful tool for the expression of some proteins; however, since we are producing a human enzyme for use in the human body, it is very important to produce the protein as a native protein as it is required for proper folding, stability, and functional activities. In other words, although the circulating half-life of drug can be improved by expressing them as a fusion protein—such as Fc-fusion and albumin-fusion—the fusion adds extra amino acids to the sequence of the target protein, which makes the protein longer than the native protein and therefore significantly negatively affects protein folding, product yield, and functional activity (Andersen et al., 2014). At this point, the expression level of ACE2-Fc fusion reported was 100 mg/kg per plant leaves. The expression levels of the truncated, soluble ACEs developed in this study are 7.5 times higher than the ACE2-Fc fusion. Both glycosylated and deglycosylated soluble ACE2 variants were purified using Ni-column affinity chromatography. The purification yield of gACE2 and dACE2 variants after the Ni-NTA single-step, were ∼0.4 and ∼0.5 g/kg per plant leaves. The purities of gACE2s or dACE2s were 90 or 95%, respectively. After size exclusion chromatography, the purity of the gACE2 and dACE2 proteins was greater than 95%. Our results showed that plant produced gACE2 and dACE2 variants successfully bind to RBD of SARS-CoV-2 spike protein. However, the dACE2 variant binds to the deglycosylated plant-produced S-protein more strongly than the glycosylated counterparts. Moreover, the plant produced gACE2 and dACE2 variants exhibited potent anti-SARS-CoV-2 activity *in vitro*. It should be note that, the IC50 values of plant produced ACE2-Fc fusion protein at the pre-entry stage was reported as 94.66 μg/ml at 25TCID50 (Siriwattananon et al., 2021). Thus, the inhibition efficiency of plant produced glycosylated, truncated form of ACE2 is ∼ 371-fold higher than that of plant produced ACE2-Fc. The IC50 values of ACE2-Fc fusion protein, produced in mammalian cells were reported as 0.1–0.8 μg/ml (Lei et al., 2020). Generally, it is supposed that the soluble form of ACE2 in excessive forms, may negatively affect the virus entering and spreading (Roshanravan et al., 2020). Based on the above descriptions, plant produced recombinant ACE2 could be used as a potential therapeutic tool in patients with COVID-19 to slow down the entry and spreading of virus and to protect the lung from damages. Similar to other respiratory diseases, COVID-19 can cause permanent damages to the lungs, heart, and other organs. A possible explanation is the blocking of the binding domain of the ACE2 receptor (RBD) by SARS-CoV-2. Therefore, recombinant ACE2 could be a promising bio-mimicking molecule attenuating and/or preventing COVID-19 related cellular injury. Thus, the development and production of a safe, functionally active, and cost-effective of recombinant ACE2 could be very useful for the treatment of patients with COVID-19. Collectively, all above findings demonstrate that plant produced glycosylated and deglycosylated forms of soluble ACE2 could be a potential therapeutic agent against SARS-CoV-2 to slow down the entry and spreading of virus and protect the lung from damages.

## Conclusion

A number of studies have shown that a recombinant ACE2 can be used as a potential therapeutic tool in patients with COVID-19. At this point, the development and production of recombinant ACE2 protein at high levels with a high anti SARS-CoV-2 activity could be a challenging task. In this study, we show that recombinant ACE2 which exhibit a potent anti-SARS-CoV-2 activity with the IC50 values of ∼1.00 μg/ml (11 nM), can be produced rapidly, at high level (∼ 0.75 g/kg plant leaf) in *N. benthamiana* plant using a plant transient expression system. These findings demonstrate that plant produced ACEs are a cost effective, safe, and promising therapeutic tool for the treatment of patients with COVID-19.

## Data Availability Statement

The original contributions presented in the study are included in the article/supplementary material, further inquiries can be directed to the corresponding author/s.

## Author Contributions

TM conceived the study. TM and AO designed the experiments. IG, MI, and DY performed the experiments. TM, GM, GH, and AO analyzed the data. TM, GM, and GH contributed to writing the manuscript. All authors reviewed the manuscript.

## Conflict of Interest

TM named inventor on patent applications covering plant produced ACE2. The remaining authors declare that the research was conducted in the absence of any commercial or financial relationships that could be construed as a potential conflict of interest.

## Publisher’s Note

All claims expressed in this article are solely those of the authors and do not necessarily represent those of their affiliated organizations, or those of the publisher, the editors and the reviewers. Any product that may be evaluated in this article, or claim that may be made by its manufacturer, is not guaranteed or endorsed by the publisher.

## Footnotes

1
https://www.sinobiological.com/recombinant-proteins/human-ace2-10108-h08h

2
https://www.activemotif.com/catalog/details/81351/recombinant-ace2-18-740-protein

3
https://www.mybiosource.com/recombinant-protein/ace2/515755

4
https://imagej.nih.gov/ij
